# Prenatal Screening Using Maternal Markers

**DOI:** 10.3390/jcm3020504

**Published:** 2014-05-09

**Authors:** Howard Cuckle

**Affiliations:** Department of Obstetrics and Gynecology, Columbia University Medical Center, 662 W 168th Street, PH1666, New York, NY 10032-3725, USA; E-Mail: hsc2121@columbia.edu; Tel.: +1-212-305-6287; Fax: +1-212-305-2262.

**Keywords:** prenatal screening, markers, spina bifida, Down syndrome, pre-eclampsia, fragile X syndrome

## Abstract

Maternal markers are widely used to screen for fetal neural tube defects (NTDs), chromosomal abnormalities and cardiac defects. Some are beginning to broaden prenatal screening to include pregnancy complications such as pre-eclampsia. The methods initially developed for NTDs using a single marker have since been built upon to develop high performance multi-maker tests for chromosomal abnormalities. Although cell-free DNA testing is still too expensive to be considered for routine application in public health settings, it can be cost-effective when used in combination with existing multi-maker marker tests. The established screening methods can be readily applied in the first trimester to identify pregnancies at high risk of pre-eclampsia and offer prevention though aspirin treatment. Prenatal screening for fragile X syndrome might be adopted more widely if the test was to be framed as a form of maternal marker screening.

## 1. Introduction

Prenatal screening is now an established part of routine antenatal care in developed countries. The common disorders being screened for include fetal neural tube defects (NTDs) such as anencephaly and spina bifida, chromosomal abnormalities such as the common trisomies, Down, Edwards and Patau syndromes, and structural abnormalities. In some countries there is also prenatal screening for single gene disorders such as cystic fibrosis (CF) and fragile X syndrome (FXS), and increasingly some are beginning to screen for maternal conditions such as pre-eclampsia. All of these activities make use of markers, which can be maternal, fetal or both, although practitioners are not always aware that that is what they are doing. The purpose of this paper is to describe the maternal markers over this range of conditions and show how they can be used optimally.

## 2. Screening and Markers

There is a fundamental difference between screening and diagnostic tests. The aim of prenatal screening is limited to the identification from among apparently healthy pregnancies those at high enough risk of a given outcome to warrant the next step: An expensive or hazardous diagnosis; another secondary screening test; or preventative action. Thus for example screening for Down syndrome does not aim to make a diagnosis of this condition, but rather to ration the use of invasive diagnostic procedures, chorionic villus sampling (CVS) or amniocentesis, that would be too hazardous, and tests that would be too expensive, to offer without prior selection.

Markers are the building blocks of screening tests; the term itself implies the lack of a definitive result that characterizes screening in comparison to diagnosis. Some prenatal markers are maternal blood analytes whilst others are bio-physical properties of the mother or the fetus. In principle, a maternal DNA profile can also be regarded as a marker although the term is not generally applied in this context. For example, finding that a woman is a CF carrier appears to be diagnostic, in so far as the carrier status of the woman has been diagnosed. However, this is misleading since being a CF carrier is not a disorder, but rather it is an indication, or marker, of increased risk of an affected pregnancy.

Many markers are continuous variables whose distribution of values is higher or lower on average in affected pregnancies. Typically, these markers have considerable overlap in the distribution of results between affected and unaffected individuals. In contrast, the distribution of values for a continuous variable used in diagnosis would have essentially no overlap. Some markers are dichotomous with a “present” or “absent” state, one of which is more common in affected compared with unaffected pregnancies. In practice, some of the continuous variables are dichotomized by grouping values but this often leads to loss of information and suboptimal performance.

A screening test can be based on a single marker or combinations of markers. It can be demonstrated statistically that the optimal way to combine markers, even when they are correlated with each other, is to estimate the risk of being affected given the marker profile. In some cases it may be possible to incorporate pre-test risk factors into the calculation. For continuous variables the choice of a cut-off level that determines whether the result is positive or negative is arbitrary as there is no intrinsic optimal cut-off that is defined by the distributions. The choice will be influenced by the relative importance of the detection rate, the proportion of affected individuals with positive results, the false-positive rate, proportion of unaffected individuals who are positive, and the positive predictive value, the chance of being affected given that the result is positive. In public health settings the false-positive rate is usually the limiting factor, since the next step is usually expensive and dominates the total cost of the screening program.

## 3. Neural Tube Defects

Perhaps the earliest example of prenatal screening is the use of a single second trimester maternal serum marker, α-fetoprotein (AFP), to identify pregnancies at high risk of anencephaly and spina bifida. In screening studies, the term “spina bifida” usually includes encephalocoele and cranial meningocoele.

An important distinction from the point of view of diagnosis is that between “open” and “closed” spina bifida. Open in this context means that there is some exposure of neural tissue or the lesion is completely covered by a thin transparent membrane, and closed means covered by skin or a thick opaque membrane. In the late 1970s, when this type of screening began, diagnosis was largely dependent on biochemical analysis of amniotic fluid and open spina bifida could be more readily diagnosed. Moreover, infants with open lesions have a poorer prognosis; about one-third will survive to 5-years and most survivors have severe handicap due to hydrocephalus, incontinence and paralysis of the lower limbs. For closed lesions about two-thirds survive to 5 years and one third of survivors have severe handicap. About one in six spina bifida lesions are closed.

Maternal serum AFP levels increase on average throughout the second trimester, doubling about every four weeks. To allow for this increase, screening programs expressed results as multiples of the gestation-specific median (MoMs) among unaffected pregnancies, using a regression equation to derive the normal median so that the exact day of gestation could be used. This method was also adopted because at that time AFP assays were not standardized and between-assay differences were proportional. The large multi-center UK Collaborative AFP Study examined levels in nearly 300 affected and almost 20,000 unaffected pregnancies [1]. The smallest overlap in the distribution of results between affected and unaffected individuals was at 16–18 weeks gestation when, on average, the AFP level was 6.4 MoM in anencephaly and 3.8 MoM in open spina bifida; closed cases had normal levels. At that gestation, using a fixed 2.5 MoM cut-off the observed detection rates were 86% for anencephaly and 76% for open spina bifida with a 3% false-positive rate. For a population with an NTD prevalence of 4 per 1000 the positive predictive value would be 1 in 10.

A large proportion of the false-positives are twins; since AFP is produced in the fetal liver, voided into the amniotic fluid and is transferred to the maternal compartment through the membranes, in normal twins the expected mean is 2 MoM. Initially, in order to have a similar false-positive rate in twins and singletons a cut-off of 5 MoM was adopted. However, most affected twins are likely to be discordant for NTDs and the detection rate will be lower than in singletons because a normal AFP mass from the unaffected co-twin will tend to mask the elevated mass from the affected twin. A compromise cut-off of 3.5 MoM was subsequently used.

Since that period there have been three developments leading to improved performance of AFP screening for spina bifida. Firstly, a negative correlation between MoM and maternal weight was found, which is usually explained in terms of dilution. A fixed mass of AFP produced by the fetal liver is diluted by a variable volume in the maternal unit. It is now standard practice to adjust for weight, dividing the observed MoM by the expected value for the weight derived by regression. Secondly, it was noted that during the second trimester the fetal head is relatively small in fetuses with spina bifida, hence using ultrasound biparietal diameter (BPD) measurement to estimate gestation is beneficial to screening. The BPD is reduced on average by the equivalent of about two weeks, systematically underestimating gestation and increasing MoMs. Furthermore modern AFP assays are considerably more precise than in the past.

Current screening performance is best estimated by statistical modelling. AFP MoMs follow an approximately log Gaussian distribution in both affected and unaffected pregnancies. The model is therefore simply characterized by three parameters: Mean MoM for spina bifida and the log standard deviations for spina bifida and normal pregnancies. The mean for spina bifida at each week of gestation can be derived by regression of the observed gestation-specific MoM from the UK Collaborative AFP Study, increased to allow for the reduced BPD—This also affects closed spina bifida. The standard deviations can be derived from the same source after reduction to allow for weight adjustment and improved assay precision. At 17 weeks the estimated detection rate is 85% for open spina bifida, 72% for open and closed lesions combined with a 1.4% false-positive rate [2].

The same 2.5 MoM cut-off will detect about nine-tenth of anencephalic cases. However, today all cases will be diagnosed by a routine 18–20 week “anomaly scan” or “genetic sonogram” (see below) and if this is not routine in a specific locality, virtually all will be picked up at a BPD dating scan. AFP screening will also detect the majority of open anterior abdominal wall defects although proportionally more cases of gastroschisis are detected than exomphalos. Generally such pregnancies are not terminated but abdominal wall defect are associated with chromosomal anomalies and the next step is to carry out a fetal karyotype. Should the fetus be chromosomally normal and the couple decides to carry on with the pregnancy, it may be felt beneficial to deliver in a unit with neonatal surgery facilities.

Fetal markers can now also be used to screen for spina bifida in the early second trimester by identification of the cranial “lemon” and cerabellar “banana” signs in the fetal skull and brain when carrying out a BPD dating scan. In spina bifida there is a tendency for the frontal bones to be scalloped or there may be absence or curvature of the cerebellum. The combined results from six retrospective studies, mainly based on examination of photographs or scans carried out when the presence of abnormality had been established, together with six prospective studies of high risk pregnancies, yielded a detection rate over 90% [3]. The false positive rate for cerebellar anomalies was negligible. Fetal ultrasound markers such as this are particularly suitable for twins, where AFP is more limited, since each fetus can be considered like a singleton.

Several first trimester ultrasound cranial markers of spina bifida have been reported, such as intracranial transluncency [4]. Whilst these may be unsuitable for screening in most centers, where there are insufficient skilled operators, a simple BPD appears to achieve high detection [5]. In spina bifida the BPD is reduced, on average, in the first as well as the second trimester. By expressing BPD in MoMs based on gestational age estimated by crown-rump length (CRL) a detection rate of 50% can be achieved for a 5% false-positive rate. In many localities amniotic fluid analysis is no longer needed for spina bifida diagnosis and referral to a tertiary center for a detailed ultrasound examination by 16 weeks gestation would suffice. Hence an even higher false-positive rate might be tolerated.

NTD prevalence varies worldwide, being much higher in populations of Celtic origin, but in recent years, dietary changes and chemo-prevention by folate supplementation or food fortification have reduced the prevalence in most developed countries. Hence the positive predictive value of screening will generally be much lower than that found in the UK during the late 1970s.

## 4. Chromosome Abnormalities

Many of the screening methods developed with NTD screening were built upon to create the, now “traditional”, programs of screening for the most frequent serious and viable chromosomal abnormality, Down syndrome. Moreover, many programs now include the other common trisomies, Edwards syndrome and Patau syndrome, and incidentally also detect other chromosome abnormalities ranging from lethal abnormalities such as triploidy to sex chromosome aneuploidies which are common but relatively benign. Some of these ideas may also eventually be incorporated in the new method of screening for chromosomal abnormalities by testing fetal cell-free (cf) DNA in maternal plasma.

The most widely used markers of Down syndrome are maternal serum human chorionic gonadotrophin (hCG), the free-β subunit of hCG, pregnancy associated plasma protein (PAPP)-A, unconjugated estriol (uE_3_), inhibin A and AFP, and ultrasound nuchal translucency (NT). The levels of all seven markers change with gestation which can be allowed for by using MoMs. For all the serum markers, the MoM values are negatively correlated with maternal weight although the extent of correlation differs between the markers. For some serum markers other co-variables of weight-adjusted MoMs have been found, including maternal smoking and ethnicity.

As with AFP in NTD screening, the distribution of all these markers follow an approximately log Gaussian distribution in both affected and unaffected pregnancies. The mean MoM in Down syndrome changes with gestation for some of the markers; this is particularly critical for NT where there is a narrow 11–13 week window when it is elevated. The standard deviations vary between localities, mainly because of differing precision in assay measurement, NT and CRL measurement. For statistical modeling the means are best estimated by meta-analysis of the literature, and the standard deviations by tailoring values in the literature to local experience [6].

The seven markers differ in the extent of overlap in the distribution of MoMs between Down syndrome and euploid pregnancies. The lack of overlap can be expressed as the absolute difference between the distribution means divided by the average standard deviation for the two distributions, a form of Mahalinobis distance. Table 1 shows this for each marker and as a guide AFP for open spina bifida has a value of about 2.9. NT is by far the single best individual marker. Among the serum markers PAPP-A has the largest Mahalinobis distance but this declines rapidly with increasing gestation. Free β-hCG is more discriminatory in the second than the first trimester and is generally more discriminatory than hCG. In the second trimester inhibin A is of comparable value to hCG.

In the mid-1980s, when AFP was first shown to be a Down syndrome marker, it was thought to only be of use in screening young women, since older women were already regarded as at high enough risk to justify amniocentesis. However, it was soon shown that the greatest detection rate for a given false-positive rate is achieved by combining, for all women, pre-test information on maternal age and family history with the AFP level to calculate the individual woman’s risk of an affected pregnancy. Risk is calculated by multiplying the pre-test risk by a factor known as the “likelihood ratio” (LR) derived from the ratio of the “heights” of the bell-shape distributions. The most reliable estimates of pre-test risk are from maternal age-specific birth prevalence rates using data collected in the period before screening became established. Using these rates, the calculated risk relates to the chance of a term pregnancy with Down syndrome. In some localities, such as the USA, it is customary to calculate the risk which relates to the chance of a mid-term affected pregnancy (some one-quarter higher) despite the lack of unbiased data on mid-term age-specific prevalence rates.

**Table 1:** Mahalinobis distance for each marker, according to gestation *.

Marker	Geatation (Weeks)	Mahalinobis Distance
NT	11	2.02
	12	1.87
	13	1.65
PAPP-A	10	1.31
	11	1.14
	12	0.90
	13	0.61
Free β-hCG (hCG)	10	0.76 (0.05)
	11	0.94 (0.32)
	12	1.05 (0.68)
	13	1.11 (1.14)
	14–18	1.33 (1.15)
AFP	14–18	0.79
uE_3_	14–18	0.83
Inhibin A	14–18	1.12

* Based on published parameters [7].

This concept was subsequently extended to incorporate multiple markers tested simultaneously or sequentially [8]. For all combinations used today the distributions of MoMs follow approximately multi-variate log Gaussian distributions with additional parameters to the single marker distributions, namely the correlation coefficients between the markers. Apart from NT all the markers are correlated to some extent. With two markers the LR is derived from the heights of the overlapping “mountains” rather than the overlapping bell-shaped distributions of a single marker. For three or more markers this is difficult to visualize but the calculation is mathematically the same. Some commercial software will calculate the risk after “truncating” the MoMs beyond certain limits where the distributions tend to deviate from Gaussian or produce counter-intuitive “risk reversal”. Some software uses two sets of distributions for Down syndrome NT values whose proportions differ according to gestational age (the so-called “mixture model”) [9].

As well as computing risks for individual women, statistical modelling can be used to predict screening performance. Indeed, model-predicted detection rates are more reliable than the observed rates in large prospective intervention studies, since these systematically overestimate detection because of “non-viability” bias. This bias arises because a proportion of those with positive screening results who have a diagnosis and termination of pregnancy would have been destined to miscarry anyway whereas non-viable affected pregnancies with negative screening results will not be known to the investigators. In addition to the multivariate log Gaussian marker distributions, the model requires a single-year maternal age distribution. Since this can vary considerably over time and between localities a standardized Gaussian distribution with mean 27 years and standard deviation 5.5 years has been proposed [10].

When multi-marker Down syndrome screening began the focus was in the second trimester using: hCG or free β-hCG and AFP (“Double” test); plus uE_3_ (“Triple” test); or both uE_3_ and inhibin A (“Quad” test). In recent years many developed countries have moved screening from the second to the first trimester using PAPP-A and either hCG or free β-hCG, together with NT (“Combined” test). The advantages of the latter include earlier diagnosis, less traumatic and safer termination of pregnancy if requested, earlier reassurance, and better screening performance. It can be done either sequentially, drawing the blood about a week before the scheduled NT scan or using a single-sample analytical analyser in a one-stop risk assessment clinic (so called, “OSCAR”).

Other sequential strategies have both first and second trimester stages. One approach is to measure PAPP-A and NT in the first trimester together with the second trimester Quad test markers (“Integrated” test) [11] or without NT (“Serum Integrated” test). These tests require the non-disclosure of first stage results until the second stage is complete which some regard as unethical, or impractical, due to the difficulty for the professional not to act on abnormal initial findings, sacrificing early diagnosis and reassurance. Alternative two-stage protocols have been suggested to overcome these limitations. One approach uses the Combined test markers as the first stage with a very high cut-off identifying less than 1% of women for CVS whilst the remainder are offered the second trimester Quad test markers and risk revision (“Step-wise” test) [12]. Another approach is similar except that only women with initial borderline negative risks, about 15%, are offered the second stage markers (“Contingent” test) [13].

When comparing different marker combinations it is best to either fix the false-positive rate and compare the detection rates or vice versa. In some countries there is a nationally agreed cut-off risk so the protocols being compared may have both different detection and false-positive rates. Table 2 shows the model predicted performance for the various screening strategies described above, according to fixed detection and false-positive rates and for a fixed cut-off. In the second trimester the use of each additional marker improves performance but first trimester screening represents a much larger incremental improvement. Two-stage sequential first and second trimester screening without NT is inferior to first trimester screening alone, but the addition of NT leads to a substantial improvement. However, there is no material advantage in testing all women in the second stage—almost equivalent performance is obtained by testing just one-sixth. All tabulated marker combinations use free β-hCG and substituting hCG leads to a loss of detection; the first trimester testing is at 11 weeks gestation, and later testing yields poorer performance (see [6]).

### 4.1. Additional Markers

Increasingly, additional markers of Down syndrome assessed at the NT scan are being incorporated into the Combined test. Three are dichotomous: Absence of the fetal nasal bone (NB); tricuspid regurgitation (TR); and absent or reversed ductus venosus (DV) blood flow. On the basis of a meta-analysis of nine studies [14], the LR for absent NB is 49 and for a present NB it is 0.31; TR has LRs of 56 and 0.44 and DV 22 and 0.35 [15]. The DV blood flow can also be assessed as a continuous variable, the pulsatility index (PI) and expressed in MoMs. This yields comparable results to the more subjective dichotomous assessment and is easier to quality control. A fourth marker, frontal-maxillary facial (FMF) angle, is also continuous but is usually dichotomized to above and below the 95th centile with LRs 45 and 0.57 [15].

**Table 2:** Down syndrome screening: Model predicted performance for different tests ^#^.

Test *	DR for FPR		FPR for DR		1 in 250 Term Risk Cut-Off		
	1%	5%	75%	85%	DR	FPR	PPV
**Second trimester**							
Double	37%	61%	12%	22%	62%	5.2%	1 in 64
Triple	42%	65%	9.9%	20%	64%	4.7%	1 in 57
Quad	50%	71%	6.9%	15%	68%	4.2%	1 in 47
**First trimester**							
Combined	74%	87%	1.2%	3.8%	81%	2.4%	1 in 23
**Both trimesters**							
Serum integrated	61%	78%	3.7%	10%	74%	3.2%	1 in 34
Integrated	85%	93%	0.3%	1.1%	87%	1.6%	1 in 15
Step-wise **	85%	94%	0.4%	1.0%	89%	1.7%	1 in 16
Contingent **	85%	92%	0.4%	1.0%	88%	1.6%	1 in 15

DR = detection rate; FPR = false-positive rate; PPV = positive predictive value; ^#^ Based on published parameters [6]; * Free β-hCG used in preference to hCG; first trimester gestation 11 weeks; ** First stage term risk cut-off 1 in 50; Contingent test borderline 1 in 50–1500.

Modelling predicts that when NB is added to the Combined test there is a substantial increase in detection (Table 3). Using DV, TR or FMF instead of NB performance is comparable although slightly less discriminatory. However, quality assurance is problematic with dichotomous variables like NB; absence is a relatively rare event, so the frequency with which the operator misclassifies this as presence or vice versa cannot be easily determined. This suggests another protocol, whereby women with borderline risks based on the Combined test are immediately referred to a specialist center for the more advanced markers [7]. This would obviate the delay associated of several weeks with a standard Contingent test and will perform almost as well (Table 3).

It is not uncommon for a woman who has had a borderline positive Combined test to delay a decision over invasive prenatal diagnosis until a second trimester “anomaly” scan has been carried out. Also some with borderline negative results may be anxious enough to require ultrasound assurance. Often the scan is interpreted simplistically, whereby the presence of a major anomaly or “soft” marker associated with Down syndrome is taken to be sufficient to tip the balance in favor of invasive testing, and the absence of signs is sufficient to contra-indicate testing. This is no longer acceptable; instead, the risk from the Combined test should be formally revised using LRs relating to each marker seen or absent [16].

In addition to this *ad hoc* use of the anomaly scan following a Combined test it could be used routinely simultaneous with the Quad test. Modeling with data from the First and Second Trimester Evaluation of Risk (FaSTER) trial [17] the performance is much better than the Quad test alone (Table 3). This assumes that risk is calculated from the serum marker profile and using, the appropriate anomaly scan LRs. Whilst this might be attractive, caution is required because of quality control considerations, particularly in the context of mass screening rather than specialist fetal medicine. This suggests contingent use of the Anomaly scan markers as with contingent NB in the first trimester and this is predicted to have a reasonable performance (Table 3).

**Table 3:** Down syndrome screening: Model predicted performance using additional markers ^#^.

Test *	DR for FPR		FPR for DR		1 in 250 Term Risk Cut-Off		
	1%	5%	75%	85%	DR	FPR	PPV
**Second trimester**							
2T-Combined	83%	93%	0.3%	1.3%	87%	1.8%	1 in 17
Quad and Anomaly scan	69%	85%	1.8%	5.2%	80%	2.9%	1 in 28
Contingent Anomaly **	67%	81%	2.2%	8.8%	76%	2.5%	1 in 26
**First trimester**							
Combined and NB	88%	95%	0.2%	0.6%	90%	1.4%	1 in 13
Contingent NB **	86%	91%	0.3%	0.8%	86%	0.9%	1 in 9
1T-Quad	44%	68%	7.6%	15%	68%	5.0%	1 in 56
Contingent NT ***	75%	85%	0.9%	4.6%	80%	2.0%	1 in 20

DR = detection rate; FPR = false-positive rate; PPV = positive predictive value; NB = nasal bone; ^#^ Based on parameters used in Table 2, published parameters for NF, NBL and PT [18], Anomaly scan markers [17] and NB [14], and parameters for PlGF based on a meta-analysis of data and studies cited in ref. [19]; * Free β-hCG used in preference to hCG; first trimester gestation 11 weeks; ** First stage term risk cut-off 1 in 50; borderline negative 1 in 51–1500; *** No first stage cut-off; borderline term risk cut-off 1 in 1500.

Many of the soft markers are qualitative so unsuitable for external quality assessment whilst others, such as nuchal skin-fold (NF), femur length (FL) and humerus length (HL) are potentially useful. On the basis of a meta-analysis of five studies where NF was expressed in MoMs, modeling predicted that incorporating this marker was increase the Double test detection rate for a 5% false-positive rate by 12% [20]. From a meta-analysis of five studies of FL and HL it has been predicted that adding NF, HL and FL would increase the Quad test detection rate for a 5% false-positive rate by 15% [21]. Another proposal is to use quantitative “facial profile” markers determined in the same plane as NF, pre-nasal thickness (PT) and nasal bone length (NBL). On the basis of a meta-analysis of five studies of PT modeling predicts that adding NF, PT and NBL to the Quad test (“2T-Combined” test) would achieve performance superior to the first trimester Combined test (Table 3) [18].

As with AFP for NTDs, multi-marker screening tests for Down syndrome are less effective in twins than singletons. Some centers will only use NT for screening twins, ideally allowing for the correlation between NT measurements between the fetuses [22,23], unless they are shown to be monochorionic, by the presence of an ultrasound placental “lamba” sign, and hence concordant. In dichorionic twins it is possible to use a Combined test although the detection rate will not be substantially better than for NT alone. However, whether or not serum markers are used the performance should be improved by using additional ultrasound markers. Large series of twins have been reported showing similar false-positive rates in twins compared with singletons for first trimester absent NB [24] and abnormal DV [25].

For localities without quality ultrasound NT facilities, a serum-only first trimester protocol might be considered. Several studies (for example [19]) have shown that maternal serum placental growth factor (PlGF) is a marker of Down syndrome in the first trimester. Second trimester markers AFP, uE_3_ and inhibin also have some discriminatory power in the first trimester [26]. Modelling predicts that a four marker protocol using serum PAPP-A, free β-hCG, PlGF and AFP (“1T-Quad” test) could yield detection comparable to standard second trimester serum only tests (Table 3).

In centers with limited equipment or operators adequately trained for NT measurement, an option is to carry out the serum screening stage of a Combined test on all women but restrict the NT stage to those who have relatively high DS risks after serum testing (“Contingent NT” test) [27]. When PlGF and AFP are also included the modelling predicts a performance similar to a standard Combined test (Table 3).

### 4.2. cfDNA Testing

All but one of the commercially available cfDNA screening tests for Down syndrome are based on counting fragments in maternal plasma, assigning each to a chromosome and quantifying the proportion assigned to chromosome 21. This proportion is a screening marker although it is not generally regarded as such, possibly because the initial aim of cfDNA testing was non-invasive prenatal diagnosis rather than screening. The results are expressed as a *z*-score computed by comparison to an expected proportion for a euploid sample. However, there are powerful co-variables that could be taken into account when interpreting the test, such as the fraction of cfDNA which is of fetal origin, the number of fragments counted and maternal weight. Allowing for these using MoMs or some other method could enhance performance [28,29]. The other commercial method uses multiplex amplification of thousands of SNP sequences which are then sequenced. The profile is assessed by maximum likelihood statistics on the probability of disomy or trisomy 21.

Seven studies of cfDNA have been carried out in maternal plasma samples taken prior to invasive prenatal diagnosis for high risk of aneuploidy [30]. The results clearly demonstrate that this is an effective secondary screening test for Down syndrome with a detection rate of 99.3% and false-positive rate less than 0.16%. Additional studies are now being reported in a more general population setting and the result are consistent with a similar performance if cfDNA were to be used routinely, as a replacement for traditional screening tests [30]. However, the test cost is too high to consider routine testing in a public health setting [31]. An alternative and much more cost-effective approach is contingent screening whereby about 15% of women with the highest Down syndrome risks based on a Combined test are selected for cfDNA testing [31,32].

### 4.3. Other Chromosomal Abnormalities

The same seven maternal serum and ultrasound markers that are used for Down syndrome screening can also be used to estimate the risk of Edwards syndrome. Some centers routinely calculate a risk and adopt an Edwards syndrome cut-off. As with Down syndrome the pre-test risk increases with maternal age and the average marker profile is similar to Down syndrome for AFP, more extreme for NT, PAPP-A and uE_3_, and in the reverse direction for hCG, free β-hCG and inhibin [6]. This means that for some protocols such as the Combined test the detection rate is very high even without explicit screening [6]. Moreover, the additional first trimester ultrasound markers such as NB can be used to improve detection of Edwards syndrome.

It has been suggested that screening is also extended to calculate a combined Edwards and Patau syndrome risk [33]. Turner syndrome, hydropic or otherwise, and triploidy, dygynic or diandric, also have their own marker profile and could also be incorporated. Ultrasound markers can also be used too, in particular fetal heart rate for Patau syndrome [34]. Furthermore, all commercial cfDNA tests now include Edwards, Patau and Turner syndromes with estimated detection rates of 97.4%, 78.9% and about 80%, with false-positive rates of 0.15%, 0.41% and 0.20% respectively, and other sex chromosome abnormalities. These additions to cfDNA testing will increase the cumulative false-positive rate to over 1%, although this may be substantially reduced when cfDNA and traditional markers are combined in a contingent test. One commercial test also includes triploidy and some tests are incorporating sub-chromosomal aneuploidy markers (“copy number variants”), those with large alleles and associated with clinically important conditions.

## 5. Cardiac Abnormalities

Serious cardiac abnormalities are identified at the 18–20 week anomaly scan but the detection rate is low. In a large European collaborative study which included 3685 malformed fetuses it was only 39% [35]. Comparable or better results are found using the first trimester ultrasound markers NT and DV. Based on a meta-analysis, a 2.5 mm cut-off for NT had a detection rate for major cardiac defects of 38% for a 4.9% false-positive rate with a positive predictive value of 0.2% and with a 3.5 mm cut-off it was 0.8% [36]. Because of these high predictive values it is worthwhile to offer fetal echocardiography to women with increased NT, regardless of the Down syndrome screening result [37]. In a meta-analysis of eight studies on euploid fetuses with NT above the 95th centile the detection rate of abnormal DV was 87% with a false-positive rate of 19% [38]. Another meta-analysis found that the performance was lower for fetuses with normal NT; 19% detection rate for a 4% false-positive rate [39]. A recent study used a combination of abnormal DV and risk of cardiac abnormality calculated from NT MoM and DV pulsatility index MoM if the DV was normal [40]. This achieved a 53% detection rate for a 4.6% false-positive rate.

## 6. Maternal-Fetal Conditions

Nicolaides has proposed that when women attend for a Combined test they are also assessed for their risk of the common maternal-fetal conditions such as pre-eclampsia, growth restriction, pre-term delivery and fetal macrosomia [41]. These conditions normally present clinically in late pregnancy, but early screening may be potentially more effective since it might facilitate primary prevention.

This concept has been most fully developed for pre-eclampsia, a complex disorder with variable presentation and outcome, but the origin is to be found in early pregnancy failure of spiral artery remodeling. In normal pregnancies, at about 8–17 weeks, trophoblasts invade the arteries, replacing the smooth muscle and endothelium. The remodeled arteries have increased blood flow and reduced resistance. When this process fails there is inadequate perfusion, placental damage, and imbalance of thromboxane-prostacyclin with consequent platelet aggregation.

Low dose aspirin has been shown to reduce the uterine artery Doppler pulsatility index [42], indicating improved blood flow [43] but until recently the consensus was that it does not prevent pre-eclampsia. However, a meta-analysis of nine published trials where treatment began before 16 weeks has led to a shift in consensus [44]. The trials included a total of 116 pre-eclampsia cases and the incidence in those assigned to aspirin was significantly reduced (relative risk 0.47, 95% confidence interval 0.34–0.65). A randomized trial of aspirin has now begun in pregnancies specifically identified as high risk through screening.

There are four first trimester markers of pre-eclampsia including maternal serum PAPP-A, which is already part of the Combined test, PlGF, which can also be used to enhance aneuploidy screening (see above), and two biophysical marker—mean arterial pressure (MAP) and uterine artery Doppler PI. MAP requires careful blood pressure measurement under standardized conditions [45]; both biophysical marker levels change with gestation so can be expressed in MoMs and the distributions are approximately log Gaussian. Another serum marker, placental protein 13, was found to be highly effective in some studies (for a review see [46]) but a reproducible commercial assay is not generally available.

As with Down syndrome screening, pre-test risk based on factors such as body mass index, parity and pre-eclampsia in a previous pregnancy or in a close family member, is modified by a LR based on the multi-marker profile. Those who were screen-positive would receive prophylactic low dose aspirin. But unlike aneuploidy, there are distinct forms of pre-eclampsia: One associated with maternal mortality and morbidity, prematurity, growth restriction and fetal death; another developing around the time of delivery, more often having a benign course, and not associated with prematurity. Some studies (for example [47]) distinguish these forms by dividing cases into “early onset”, requiring delivery before 34 weeks, and “late onset”, while recognizing that this is merely an operational rather than definitive indicator of severity.

The best estimate of screening performance can be derived from the large population based study at Kings College Hospital, London [47]. For a 5% false-positive rate, the model predicted early pre-eclampsia detection rate is 78%. Since the next step is simply aspirin prophylaxis, which is neither expensive nor, with a few exceptions, particularly hazardous, it can be argued that a 10% false-positive rate is acceptable in which case the detection rate would increase to 87%. For late onset pre-eclampsia the predicted detection rate is lower: 70% for those delivering at 34–37 weeks and 48% later. Early iatrogenic delivery because of pre-eclampsia and spontaneous preterm delivery will both influence the actual gestation of delivery in an individual pregnancy. A model (so called “Competing risks”) has been proposed to calculate the chance of pre-eclampsia before a given gestation, taking into account the chance of a spontaneous delivery before that date. The estimated performance of pre-eclampsia screening is greater using this model [48].

The aspirin meta-analysis also showed that early treatment reduces the risk of growth restriction. And pregnancies at high risk of this disorder, in the absence of pre-eclampsia, can be identified using all the aneuploidy and pre-eclampsia markers in combination, including NT and hCG [49].

## 7. Single Gene Disorders

Fragile X syndrome (FXS) is the most frequent cause of severe learning disability after Down syndrome and the main inherited cause. Most affected males have moderate-profound disability whilst females are often less severely affected. It has a non-Mendelian pattern of inheritance and since 1991 when the gene for FXS was characterized, the pattern was explained [50]. At the gene locus, FMR1, mutation involves hyper-expansion of a repeat CGG sequence. A mild expansion of 55–199 repeats, or premutation (PM), when transmitted by a female carrier may be further expanded to 200+ repeats, or full mutation (FM), with methylation of an adjacent CpG island causing loss of expression. All males with a FM have FXS but only half of the females with a FM on one of their X chromosomes are clinically affected.

Considering an increased repeat sequence as a marker of FRX it is possible to calculate for each screened women the risk of an affected pregnancy. The chance of the fetus receiving a specific copy of chromosome X is unrelated to the repeat size and there are published curves for the chance of a given PM expanding to an FM. Using this information and the observed PM frequency in different screening studies it is simple to estimate the detection rate with a 55 repeat cut-off as close to 100%, the false-positive rate 0.4% and positive predictive value close to 5% [51].

Pre-test counselling is more difficult than with NTD or Down syndrome screening. Firstly, in a female FM carrier fetus there is no way of knowing whether FXS is present, although in prospective intervention screening studies almost all such pregnancies were terminated (for example, [52]). Secondly, female PM carriers are at risk of a late onset neurodegenerative disorder—in one study 8% after age 40 [53]—and male PM carrier fetuses are at even higher risk. About one-quarter of female PM carriers also experience a decline in ovarian function leading to early menopause. Whilst the latter may be considered a downside of screening it could also be seen positively as a means of informing future reproductive choices.

Hemoglobinopathies, β-thalassaemia and sickle cell disease are common is some countries and largely confined to ethnic subgroups in others. Carriers can be detected through screening for red cell indices and the characterization of hemoglobin variants. For example, mean corpuscular hemoglobin is routinely determined during pregnancy in many countries and can be used as a screening marker.

Recessive conditions such as cystic fibrosis (CF) and spinal muscular atrophy (SMA) are relatively common, the former particularly so in North European populations. Nevertheless, antenatal screening programs have not been established in most countries, although multi-disorder protocols such as the Ashkenazi-Jewish Panel have been developed for sub-populations. The aim of prenatal screening is to identify carrier couples rather than individual carriers since the chance of an affected fetus in a given pregnancy is 1 in 4. A contingent strategy is most efficient: Test the mother using blood drawn for other antenatal tests and test the father only if she is found to be a carrier. This is best done by obtaining a saliva sample from the father in advance of the maternal test.

Perhaps there would be more public health interest in such screening if being identified as a carrier was regarded as a marker rather than an entity in itself. Performance can be predicted from the carrier frequency and the proportion of carriers that can be identified. To illustrate this consider the example of UK Caucasians who have a CF carrier frequency of about 4% and multiple-mutation gene analysis can identify about 85% of disease causing mutations in the *CFTR* gene of an individual [54]. In this population 0.16% (4% × 4%) of couples will both be CF carriers and screening will detect 72% (85% × 85%) of such couples. With the marker approach a “positive” screening test means that the parents both have markers, *i.e.*, they are identified as CF carriers. As all CF pregnancies are in carrier couples the screening detection rate will also be 72%. For each pregnancy the screening total positive rate will be 0.12% (0.16% × 72%) and since 3 in 4 pregnancies to carrier couples are unaffected the false-positive rate will be approximately 0.09% (0.12% × 75%).

## 8. Conclusions

Maternal marker screening has advanced rapidly since AFP was first used to screen for NTDs. There are now a range of high performance tests available to screen for chromosomal abnormalities suiting localities with different financial restraints and availability of quality ultrasound. Although cfDNA testing is still too expensive to be considered for routine application in public health settings, it can be cost-effective when used in combination with traditional markers. The latter can be built upon by the addition of markers for non-chromosomal disorders and pregnancy complications. Prenatal screening for single gene disorders might be adopted more widely if the tests were considered as maternal marker screening.
